# Temporal Bone Fractures on High-Resolution CT: Bridging Radiologic Detail with Otologic Anatomy and Surgical Implications

**DOI:** 10.3390/diagnostics16050718

**Published:** 2026-02-28

**Authors:** Osama M. K. Edris, Abdulgaffar Bashir Adam, Emad Ali Albadawi, Ahmad Mahroos ALGhabban, Razan Saad M. Alqarni, Wejdan Hussain Owaydhah, Omar A. Alharthi, Eyad Khattab, Fahd Alharbi, Yasir Hassan Elhassan

**Affiliations:** 1 Department of Otolaryngology, Faculty of Medicine, University of Khartoum, Khartoum 11115, Sudan; osamamk@yahoo.com; 2 Khartoum ENT Teaching Hospital, Khartoum 11112, Sudan; abdomma77@gmail.com; 3 Department of Basic Medical Sciences, College of Medicine, Taibah University, Madinah 42353, Saudi Arabia; ebadawi@taibahu.edu.sa (E.A.A.); rqarni@taibahu.edu.sa (R.S.M.A.); wawedh@taibahu.edu.sa (W.H.O.); 4 Department of Internal Medicine, College of Medicine, Taibah University, Madinah 42353, Saudi Arabia; amghabban@taibahu.edu.sa (A.M.A.); oharthe@taibahu.edu.sa (O.A.A.); 5 Department of Emergency Medicine, King Saud University Medical City, Riyadh 42353, Saudi Arabia; eykhattab@ksu.edu.sa; 6 Department of General and Specialized Surgery, College of Medicine, Taibah University, Madinah 42353, Saudi Arabia; f.aalharbi@taibahu.edu.sa

**Keywords:** temporal bone fractures, high-resolution computed tomography, otologic trauma, craniofacial fractures

## Abstract

**Primary Objective**: To characterize high-resolution computed tomography (HRCT) fracture patterns, namely orientation and otic capsule status, among Sudanese patients with acute temporal bone trauma. **Secondary Objectives:** (i) To quantify the prevalence and pattern of concomitant craniofacial fractures, (ii) to describe early audiologic outcomes, and (iii) to document facial nerve dysfunction. **Methods**: Prospective cross-sectional study of 45 consecutive patients (≥5 years) with HRCT-confirmed TBF sustained within 7 days of injury, managed at two tertiary otolaryngology centers in Khartoum (October 2022–March 2023). All imaging, clinical, and audiologic variables were recorded once at the index presentation (≤7 days after trauma); the study did not include longitudinal follow-up. Two blinded experts independently classified fracture orientation (longitudinal, transverse, mixed/oblique), otic capsule status (sparing [OCS] vs. otic capsule-violating [OCV]), and ancillary HRCT signs (ossicular chain disruption, tympanic plate fracture, pneumolabyrinth/CSF leak); inter-observer reliability was assessed with Cohen’s κ. Concomitant craniofacial fractures, pure-tone audiometry, and House–Brackmann facial nerve grades were recorded. Predictor–outcome associations were examined with χ^2^ statistics (*p* < 0.05). **Results**: Mean age 35.9 ± 17.4 years; 78% male. Road traffic accidents were associated with 58% of injuries. HRCT showed 60% longitudinal, 20% transverse, and 20% mixed/oblique fractures; 27% were OCV. Ossicular chain disruption, tympanic plate fracture, and ppneumolabyrinthCSF leak were present in 17.8%, 13.3%, and 8.9%, respectively. Concomitant craniofacial fractures occurred at 27%, chiefly Lefort III (15.6%) and Lefort II (8.9%). Transverse/mixed fractures were strongly associated with Lefort II–III injuries (χ^2^ = 16.2, *p* = 0.001); age (*p* = 0.21) and sex (*p* = 0.08) were non-significant. Conductive, sensorineural, and mixed hearing loss affected 69%, 13%, and 18%; facial nerve palsy occurred in 58%. Inter-observer agreement was substantial to almost perfect for all imaging variables (κ = 0.77–0.92). **Conclusions:** Although longitudinal fractures predominated, over one-quarter breached the otic capsule and one-fifth followed transverse/mixed planes, configurations associated with higher odds of conductive deafness, facial nerve palsy, and complex mid-facial fractures. HRCT provides reliable characterization and should underpin comprehensive head-and-mid-face trauma protocols. Enhanced road safety policies and multidisciplinary trauma care are vital for reducing neuro-otologic morbidity in resource-limited settings.

## 1. Background

Head and neck trauma constitutes a substantial global health burden, with road traffic collisions, interpersonal violence, and accidental falls accounting for a large proportion of emergency presentations [1,2]. Among all skull fractures, the temporal bone is involved in 3–22% of cases [3]. Although 80–90% of temporal bone fractures are unilateral, their consequences can be profound, encompassing conductive or sensorineural hearing loss, facial nerve palsy, vestibular dysfunction, and cerebrospinal fluid (CSF) leakage [4].

**Figure 1:**
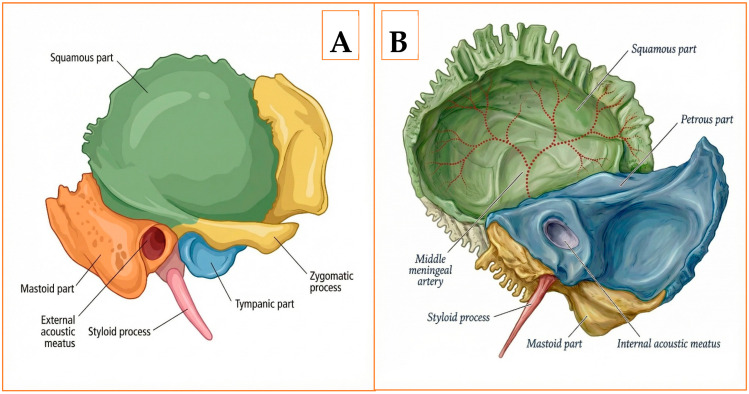
Anatomy of the right temporal bone. (**A**) Lateral external (otologic) surface. (**B**) Medial intracranial surface.

The temporal bone is composed of four distinct segments—squamous, mastoid, tympanic, and petrous (Figure 1A,B). The petrous portion is clinically pivotal because it encloses the otic capsule, the facial nerve canal, and the intrapetrous segment of the internal carotid artery [5]. Forming the medial wall of the middle ear and the anterior boundary of the posterior cranial fossa, this pyramidal mass also transmits the facial and vestibulocochlear nerves through the internal acoustic meatus and provides bony support for the cochlea and semicircular canals [6,7]. Consequently, any traumatic breach of the petrous ridge is associated with an increased likelihood of sensorineural hearing loss, facial nerve dysfunction, and vascular injury, reinforcing the need for meticulous multidetector HRCT assessment in head-injured patients [8,9].

**Figure 2:**
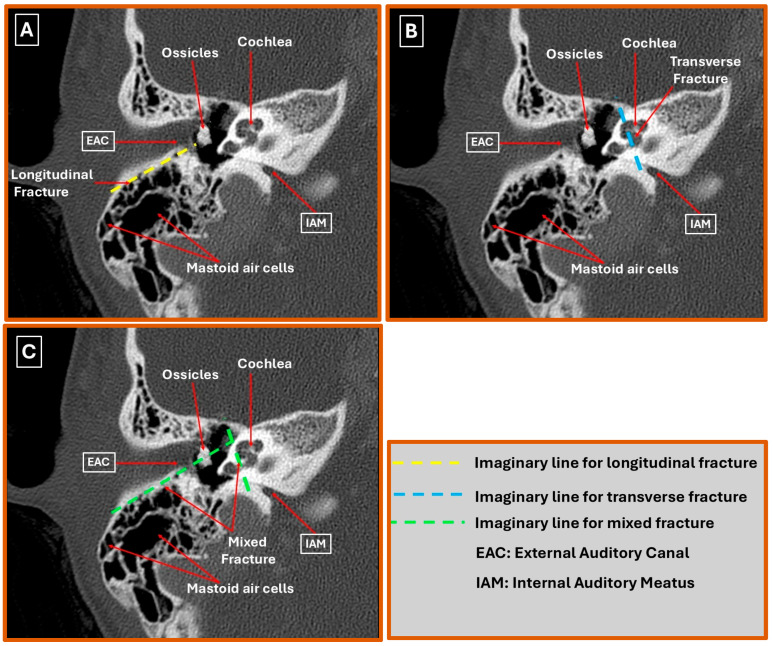
High-resolution CT patterns of temporal bone fractures in the right ear. (**A**) Longitudinal fracture: Lucent line parallels the petrous axis from the external acoustic meatus (EAM), sparing the otic capsule. (**B**) Transverse fracture: High-attenuation line traverses the petrous pyramid perpendicularly, breaching the internal acoustic meatus (IAM) and cochlear labyrinth (otic capsule-violating). (**C**) Mixed/oblique fracture: Intersecting fracture lines produce a stellate configuration that crosses both squamous and petrous cortices with associated ossicular dislocation and tympanic plate disruption.

Three principal fracture orientations can be distinguished on high-resolution CT (HRCT) of the injured temporal bone (Figure 2A–C). Longitudinal fractures—generated predominantly by lateral impact—run parallel to the petrous axis and usually propagate along the squamosal mastoid junction while sparing the otic capsule; HRCT typically demonstrates a low-attenuation line extending from the external acoustic meatus toward the foramen lacerum, often with hemotympanum or ossicular displacement [2,10]. Transverse fractures, produced by high-energy anteroposterior forces, cross the petrous pyramid perpendicularly, intersect the internal acoustic meatus and labyrinth, and are strongly associated with otic capsule-violating (OCV) patterns—appearing on HRCT as sharp, high-attenuation lines traversing the bony labyrinth, frequently accompanied by pneumolabyrinth or perilymphatic fistula [11,12]. Mixed/oblique trajectories combine elements of both and create complex fracture geometries; HRCT may reveal stellate lines that breach squamous and petrous cortices and show a higher prevalence of ossicular chain disruption or tympanic plate fractures [7,13]. Because fracture orientation and otic capsule status are directly associated with the risks of sensorineural hearing loss, facial nerve palsy, and cerebrospinal fluid leakage, careful multiplanar reformats—especially coronal oblique and Stenvers-type reconstructions—remain indispensable for comprehensive injury mapping [14,15].

High-resolution CT (HRCT) is the current gold standard for evaluating temporal bone trauma. Modern multidetector protocols reliably define fracture orientation, ossicular chain disruption, otic capsule violation, and other occult complications [8,9,13]. Although Ulrich’s traditional longitudinal versus transverse scheme remains clinically useful, it does not encompass the full spectrum now evident on HRCT [10]. The more recent otic capsule-sparing (OCS) versus otic capsule-violating (OCV) classification correlates better with hearing outcome, facial nerve injury, and risk of CSF leak [11]. Nonetheless, fractures of the tympanic plate are still frequently overlooked [14,15], and missed diagnoses can lead to external auditory canal stenosis, trismus, and chronic otorrhoea—reinforcing the need for meticulous HRCT review.

Temporal bone injury seldom occurs in isolation; up to one-quarter of patients with blunt head trauma harbor concomitant skull base or mid-facial fractures that complicate airway management, lengthen hospital stay, and worsen prognosis [16,17]. Precise knowledge of common fracture constellations, therefore, helps emergency physicians and otologic surgeons anticipate structures at risk and plan timely interventions.

Despite these clinical imperatives, most published data originates from high-income countries with established trauma systems. Evidence from low- and middle-income regions is scarce, and no Sudanese study has yet detailed the HRCT characteristics of temporal bone fractures or quantified their association with other craniofacial injuries. This information gap limits context-specific preventive strategies and may delay multidisciplinary management in resource-constrained settings where advanced neuro-otologic care is limited.

### Aim

The primary aim of this study was to characterize HRCT-defined temporal bone fracture patterns, specifically fracture orientation and otic capsule status, in patients presenting with acute otologic trauma. Secondary aims were (i) to quantify the prevalence and distribution of concomitant craniofacial fractures, and (ii) to describe early audiologic outcomes and facial nerve function in the same cohort.

## 2. Methods

### 2.1. Study Design and Setting

A descriptive cross-sectional study was undertaken between 1 October 2022 and 31 March 2023 at two tertiary referral centers in Khartoum, Sudan: Khartoum ENT Teaching Hospital and Ibrahim Malik Teaching Hospital. Both institutions provide 24 h emergency otorhinolaryngology services and receive patients from across the country.

All demographic, clinical, audiologic, and radiologic data were captured prospectively during each patient’s initial assessment within seven days of injury; no serial or long-term follow-up measurements were obtained, underscoring that this was a cross-sectional (not longitudinal) investigation.

### 2.2. Participants

All consecutive patients presenting clinical evidence of ear trauma who underwent high-resolution computed tomography (HRCT) of the temporal bone during the study period were screened for eligibility.

Inclusion criteria:
  - Age ≥ 5 years (to ensure reliable audiometry);
  - HRCT-confirmed temporal bone fracture related to acute ear/head trauma;
  - Presentation within 7 days of injury;
  - Provision of written (or guardian) informed consent.
Exclusion criteria:
  - Ear trauma without radiologic evidence of temporal bone fracture;
  - Incomplete clinical, audiologic, or CT documentation;
  - Previous temporal bone surgery or chronic ear disease that could confound imaging interpretation.

### 2.3. Sample Size

A total coverage strategy was adopted because of the limited case load; all 45 eligible patients during the 6-month window were enrolled.

### 2.4. Data Collection Procedures

A pre-tested, interviewer-administered questionnaire captured demographic data (age, sex, occupation), injury mechanism, and presenting symptoms. Systematic otologic and neurologic examinations were performed by an otolaryngology resident under the supervision of a consultant. Audiologic assessment included:

1. Tuning fork tests (256 Hz and 512 Hz) for Rinne and Weber responses.
2. Pure-tone audiometry (PTA) was performed in a sound-treated booth using a clinical audiometer (AC40, Interacoustics A/S, Middelfart, Denmark), measuring air- and bone-conduction thresholds across 0.25–8 kHz. Hearing loss was classified as conductive, sensorineural, or mixed based on air-bone gaps. PTA thresholds were averaged across 0.5, 1, 2, and 4 kHz (four-frequency PTA). Hearing categories were defined as follows: (i) conductive hearing loss (CHL)—air-bone gap ≥ 15 dB with bone-conduction PTA ≤ 25 dB HL; (ii) sensorineural hearing loss (SNHL)—bone-conduction PTA > 25 dB HL with air-bone gap < 15 dB; and (iii) mixed loss—bone-conduction PTA > 25 dB HL together with an air-bone gap ≥ 15 dB. Severity strata (mild, moderate, severe, profound) were not applied because early post-traumatic thresholds can fluctuate.
  PTA was attempted in all 45 participants within 48 h of presentation; 42 (93%) completed testing successfully. The three incomplete tests (two children < 6 years; one hemodynamically unstable adult) were excluded from quantitative PTA analyses but retained in descriptive symptom counts. No data imputation was performed.
3. Facial nerve function was graded using the House–Brackmann system and examined by an otologist within the first 24 h of hospital admission and before any therapeutic intervention. Palsy present on initial examination was classified as immediate onset, whereas new or worsening dysfunction documented during the first 7 days of observation was recorded as delayed onset.

### 2.5. Imaging Protocol

All patients underwent HRCT of the temporal bone on a 64-slice multidetector scanner (GE Optima™ CT660, GE Healthcare, Wauwatosa, WI, USA) using the following parameters: 0.6 mm collimation, 120 kVp, 250 mAs, pitch 0.9, and a high-frequency reconstruction algorithm. Axial images were acquired parallel to the infra-orbito-meatal line; coronal and sagittal reformats (0.6 mm thickness) were generated on an AW workstation, version 4.7 (GE Healthcare, Wauwatosa, WI, USA). In every case, the acquisition extended from the cranial vertex to the mandibular inferior border, thereby providing a dedicated head-and-mid-face data set for systematic evaluation of associated craniofacial fractures. All fracture classifications were performed on the full multiplanar data set, i.e., native axial images together with standard coronal and sagittal reformats plus oblique Posch and Stenvers planes, thereby ensuring optimal visualization of the otic capsule and facial canal [9,13]. Images were reviewed independently by a senior radiologist and an otologist, with disagreements resolved by consensus.

### 2.6. Operational Definitions

Cerebrospinal fluid (CSF) leak was defined by at least one of the following:
  - persistent clear otorrhoea or rhinorrhea with a positive β_2_-transferrin or β-trace-protein assay (performed in 6 cases where laboratory facilities were available);
  - radiologic evidence of pneumolabyrinth or air within the middle-ear/mastoid cavity on HRCT;
  - intra-operative confirmation of perilymphatic or CSF egress.
Hearing-loss classification: Categories were defined as follows:
  - Conductive hearing loss (CHL): Air–bone gap ≥ 15 dB with bone-conduction four-frequency PTA ≤ 25 dB HL.
  - Sensorineural hearing loss (SNHL): Bone-conduction four-frequency PTA > 25 dB HL with air–bone gap < 15 dB.
  - Mixed hearing loss: Bone-conduction four-frequency PTA > 25 dB HL together with an air–bone gap ≥ 15 dB. (Severity strata were not applied because early post-traumatic thresholds can fluctuate.)

Audiologic timing and completeness: Pure-tone audiometry (PTA) was attempted in all patients within 48 h of presentation using a calibrated booth-based audiometer. Forty-two of 45 patients (93%) completed PTA; the remaining 3 were either < 6 years of age (n = 2) or clinically unstable (n = 1). These three cases were excluded from quantitative audiometric analyses but retained in symptom-frequency calculations. No imputation of missing audiometric data was undertaken.

### 2.7. Inter-Observer Reliability

Two independent readers, a fellowship-trained neuroradiologist (15 years of experience) and an otologist (12 years of experience), reviewed all HRCT examinations in a blind fashion. Both readers had access only to de-identified image sets and were blinded to all clinical information, including patient demographics, injury mechanism, audiologic findings, and facial nerve status; only the side of the suspected injury (left/right/bilateral) was provided to focus their attention on the relevant temporal bone. For every scan, they coded: (i) fracture orientation (longitudinal/transverse/mixed/oblique), (ii) otic capsule status (OCS vs. OCV), (iii) ossicular chain disruption (present/absent), and (iv) tympanic plate fracture (present/absent). After independent scoring, ratings were compared, and Cohen’s κ coefficients with 95% confidence intervals (CI) were calculated to quantify inter-observer agreement. Discrepancies were resolved by joint consensus to generate the final analytic dataset. Before formal scoring began, the two observers conducted a 30 min calibration session during which they jointly reviewed five pilot cases (not included in the study cohort) to harmonize the application of fracture orientation, otic capsule status, and ancillary findings criteria. No additional consensus training sessions were held once independent scoring commenced. κ values were interpreted according to contemporary benchmarks, whereby < 0.20 indicates “slight,” 0.21–0.40 “fair,” 0.41–0.60 “moderate,” 0.61–0.80 “substantial,” and > 0.80 “almost perfect” agreement [17].

Fractures were categorized by:

- Classical orientation—longitudinal, transverse, or mixed/oblique [10].
- Otic capsule status—otic capsule-sparing (OCS) or otic capsule-violating (OCV) [3].
- Presence of tympanic plate disruption, ossicular dislocation, facial canal involvement, pneumolabyrinth, or CSF leak.

Associated craniofacial fractures (Lefort I–III, zygomatic, orbital floor, mandibular, etc.) were concurrently recorded.

### 2.8. Study Variables and Outcomes

Independent variables: Age, sex, mechanism of injury, and laterality.

Primary outcome: HRCT fracture pattern (classical orientation and otic capsule status). Secondary outcomes: (i) Presence and type of concomitant craniofacial fractures; (ii) pure-tone audiometry category (conductive, sensorineural, mixed); and (iii) House–Brackmann facial nerve grade.

### 2.9. Statistical Analysis

Data were entered into IBM SPSS Statistics version 26.0 (IBM Corp., Armonk, NY, USA). Categorical variables are expressed as frequencies and percentages; continuous variables as means ± standard deviations (SD) or medians (inter-quartile range) where appropriate. Associations between fracture pattern (longitudinal/transverse/mixed/oblique; OCS/OCV) and categorical predictors were tested with Pearson’s χ^2^ or Fisher’s exact test. A two-sided *p* < 0.05 was considered statistically significant. Given the modest sample size, the analyses were conceived as exploratory; consequently, only univariate comparisons (Pearson’s χ^2^ or Fisher’s exact test, as appropriate) were performed, and all reported associations should be interpreted as non-confirmatory. Where cell counts permitted, crude odds ratios (ORs) with 95% confidence intervals (CIs) were calculated to complement *p*-values and enhance clinical interpretability.

## 3. Results

### 3.1. Primary Outcome: HRCT Fracture Patterns

**Figure 3:**
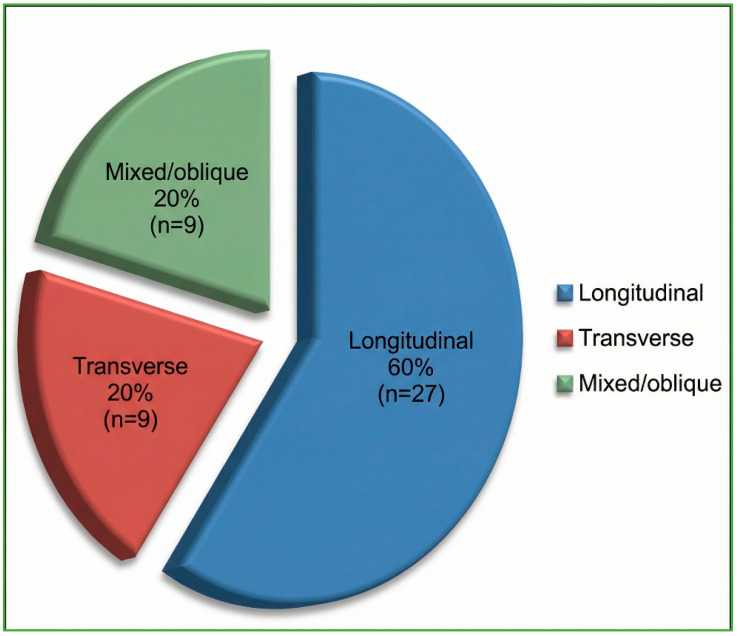
HRCT distribution of temporal bone-fracture orientations (n = 45). Percentages are rounded to whole numbers; longitudinal = 60%, transverse = 20%, mixed/oblique = 20%.

**Figure 4:**
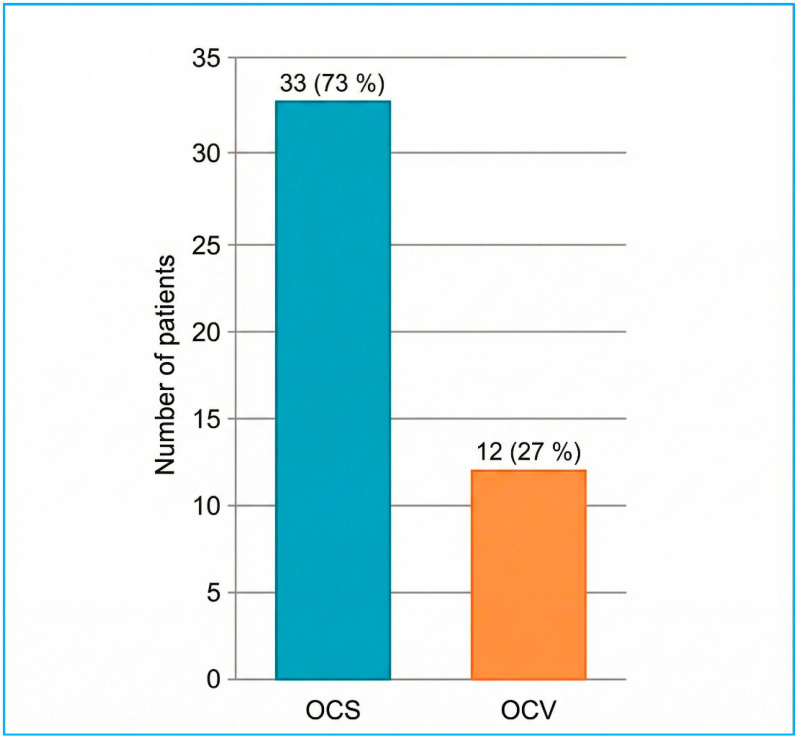
Otic capsule status of temporal bone fractures on HRCT (n = 45). OCS = otic capsule-sparing; OCV = otic capsule-violating.

The distribution of fracture orientations and otic capsule status, predetermined as this study’s primary outcome, is presented first. Classical fracture orientation on high-resolution CT was longitudinal in 27/45 cases (60%), transverse in 9/45 (20%), and mixed/oblique in 9/45 (20%) (Figure 3). According to the otic capsule paradigm, 33/45 fractures (73.3%) spared the capsule (OCS), whereas 12/45 (26.7%) violated it (OCV) (Figure 4). Inter-observer agreement for orientation and otic capsule status was almost perfect (κ = 0.86 and 0.92, respectively).

### 3.2. Demographic Profile and Injury Mechanism

**Figure 5:**
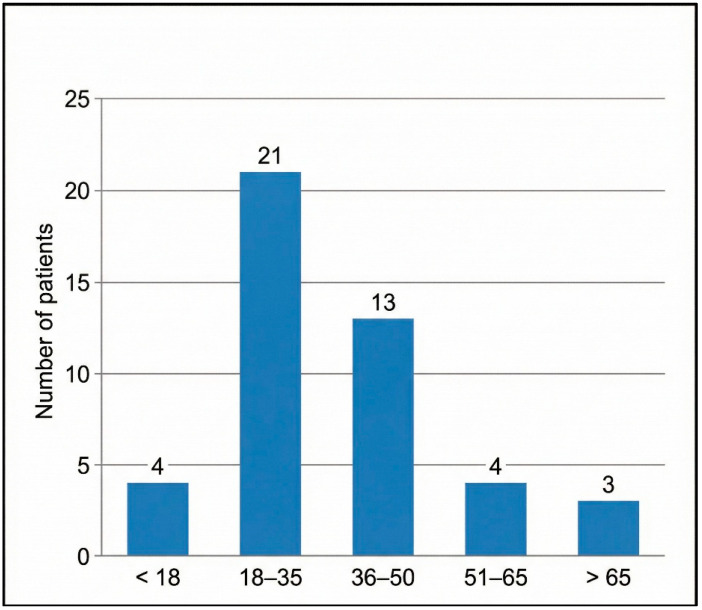
Age distribution of patients with HRCT-confirmed temporal bone fractures (n = 45).

The cohort was predominantly young adults (mean age ± SD = 35.9 ± 17.4 years) and male (male-to-female ratio = 3.5:1). Road traffic accidents (RTAs) were the main mechanism of injury (58%), followed by interpersonal blows (18%). Detailed frequencies are given in Table 1, and the age distribution is depicted in Figure 5.

Bar heights indicate the absolute number of patients in each predefined age category. Age groups were chosen a priori to align with WHO adult age strata for trauma reporting.

### 3.3. Clinical Presentation, Otoscopic, and Neurologic Signs

Decreased hearing was the dominant symptom (80%), followed by otorrhoea (44%), tinnitus (42%), and vertigo (33%). Objective examination revealed an external auditory canal (EAC) laceration in 36% of patients and a tympanic membrane perforation in almost half. Facial nerve palsy (House–Brackmann ≥ II) was present in 58%. Of the 26 palsies, 19 (73%) were immediate onset, and seven (27%) were documented as delayed onset during inpatient follow-up. Pure-tone audiometry showed conductive hearing loss (CHL) in 69%, sensorineural hearing loss (SNHL) in 13%, and mixed loss in 18% (Table 2). Complementary HRCT findings for the same cohort are summarised in Table 3.

### 3.4. Secondary Outcomes: Craniofacial Injuries, Audiologic, and Facial Nerve Findings

The following paragraphs summarize the three prespecified secondary outcomes of the study.

#### 3.4.1. Concomitant Craniofacial Fractures

Associated craniofacial fractures were prospectively recorded after a systematic review of the full head-and-mid-face HRCT dataset in all 45 patients. Fractures were detected in 12/45 patients (27%), predominantly Le Fort III (7/45, 15.6%) and Le Fort II (4/45, 8.9%) patterns (Table 4). No isolated zygomatic, orbital floor, or mandibular fractures were recorded.

#### 3.4.2. Audiologic Outcomes

As noted above, CHL was the most frequent deficit (31/45, 68.9%), followed by SNHL (6/45, 13.3%) and mixed loss (8/45, 17.8%).

#### 3.4.3. Facial Nerve Function

Early House–Brackmann grading was completed within 24 h of presentation. Facial nerve palsy (HB ≥ II) occurred in 26/45 patients (57.8%), with 19 immediate-onset and 7 delayed-onset cases.

### 3.5. Inter-Observer Agreement

Agreement between the neuroradiologist and otologist was high across all key variables. For fracture orientation, observed agreement was 91% (41/45 cases) with κ = 0.86 (95% CI, 0.72–1.00), indicating almost-perfect concordance. Otic capsule status showed 96% agreement (κ = 0.92; 95% CI, 0.80–1.00). Substantial agreement was recorded for ossicular chain disruption (89%, κ = 0.77; 95% CI, 0.55–0.99) and tympanic plate fractures (93%, κ = 0.84; 95% CI, 0.66–1.00).

### 3.6. Inferential Analysis

In exploratory univariate testing, fracture orientation (longitudinal vs. non-longitudinal) was not associated with age group (*p* = 0.21) or sex (*p* = 0.08). Non-longitudinal fractures demonstrated markedly higher odds of concomitant Le Fort II–III injury (OR = 8.4, 95% CI 2.0–34.6; χ^2^ = 16.2, *p* = 0.001). The full set of χ^2^ results is summarized in Table 5. Crude effect-size estimates underscore these findings: non-longitudinal fractures carried eight-fold higher odds of a concomitant Le Fort II–III injury (OR = 8.4, 95% CI 2.0–34.6; χ^2^ = 16.2, *p* = 0.001), otic capsule-violating fractures showed nearly five-fold higher odds of a cerebrospinal fluid leak (OR = 5.1, 95% CI 0.8–33.6), and they were associated with a three-and-a-half-fold increase in early facial nerve palsy (OR = 3.6, 95% CI 1.0–13.0). All odds ratios for facial nerve palsy refer to the combined cohort of immediate + delayed cases, as defined in Section 2.4**.** A complete list of crude odds ratios with 95% confidence intervals for all key 2 × 2 comparisons is provided in Supplementary Table S1.

Detailed cross-tabulation of otic capsule status versus hearing-loss type and facial nerve palsy is provided in Supplementary Table S2

- Fracture orientation was dichotomized as longitudinal versus non-longitudinal (transverse or mixed/oblique) to satisfy the minimum-cell assumption of the χ^2^ test.
- “Concomitant craniofacial fracture” refers to any Lefort I–III mid-facial fracture identified on the same HRCT study.

### 3.7. Key Findings

- Longitudinal fractures were the most prevalent (60%), whereas one in four fractures violated the otic capsule.
- Conductive hearing loss was the dominant audiologic outcome (69%).
- Additional craniofacial fractures were present in 27% of patients, predominantly Lefort III patterns.
- Transverse and mixed/oblique fractures demonstrated a significant association with high-grade mid-facial (Lefort II–III) fractures (*p* = 0.001), whereas age and sex were not correlated with fracture orientation.

## 4. Discussion

This study’s primary finding is that high-resolution computed tomography (HRCT) can reliably classify temporal bone fractures by both fracture orientation and otic capsule status in a resource-limited environment. To our knowledge, this hospital-based investigation is also the first Sudanese series to provide such a detailed HRCT appraisal of temporal bone fractures (TBFs) and their craniofacial accompaniments, from which four overarching messages emerge. First, longitudinal fractures predominated (60%), yet more than one-quarter (27%) breached the otic capsule—a configuration that has been associated with increased neuro-otologic morbidity in prior work. Second, conductive hearing loss and facial nerve palsy were the commonest early sequelae, affecting 69% and 58% of patients, respectively. Third, non-longitudinal (transverse or mixed/oblique) fractures showed a strong association with complex mid-facial (Lefort II–III) injuries, whereas patient age and sex were not predictive. Finally, road traffic accidents (RTAs) accounted for almost three-fifths of cases, suggesting—but not proving—that high-energy mechanisms are common among patients who reach tertiary ENT services in our setting. Furthermore, the reliability of our imaging assessments was excellent: inter-observer agreement between the neuroradiologist and otologist was substantial to almost perfect across all key variables (κ = 0.77–0.92), confirming the robustness and reproducibility of our HRCT-based classifications [18].

### 4.1. Comparison with Previous Work

Fracture patterns in the present cohort align closely with global experience. The 60% prevalence of longitudinal fractures echoes the 70–80% reported from high-income trauma registries [14,18]. Our 20% rate of transverse fractures also falls within the 10–30% range observed in large CT-based series [5,10]. The proportion of otic capsule-violating (OCV) injuries exceeded several Western reports (12–25%) [3], and may be related to the high RTA burden (58%) in Khartoum. Two further factors could amplify this proportion. First, road traffic accidents in our setting often involve high-velocity mechanisms (e.g., unrestrained occupants, motorcycle–vehicle collisions) that transmit greater anteroposterior forces to the petrous apex, a vector well known to favor labyrinthine disruption. Second, both participating hospitals are tertiary ENT referral centers; less severe, capsule-sparing fractures managed in peripheral units are therefore under-represented, enriching our sample for complex OCV injuries. Contemporary multidetector CT investigations have confirmed that the anteroposterior forces typical of vehicular collisions propagate energy across the skull base, fostering OCV and mixed-plane fractures [13,19].

Regarding functional outcome, the 69% rate of conductive hearing loss parallels the 60–75% described by Juliano [7] and by Deshmukh et al. [20]. Sensorineural loss (13%) mirrors earlier HRCT series linking profound deficits chiefly to otic capsule disruption [13,15]. Our facial nerve-palsy prevalence (58%) substantially exceeds the 15–25% recorded in temperate-region tertiary centers [11,21]. Several factors probably account for this disparity. First, our definition of facial nerve dysfunction encompassed all deficits of House–Brackmann grade II or higher; inclusion of mild, often transient, weakness is known to raise overall prevalence figures. Second, HB grading was performed within 24 h of injury, when perineural edema may mimic or exaggerate permanent structural damage. Third, both participating hospitals function as supra-regional ENT referral hubs: patients with minor head trauma are typically managed in peripheral facilities and never undergo temporal bone CT, whereas those with more severe or complicated injuries—among whom facial palsy is over-represented—are preferentially transferred, thereby inflating the observed rate. However, the cross-sectional design precludes a definitive causal conclusion.

The observed linkage between transverse/mixed fractures and Lefort II–III patterns (χ^2^ = 16.2, *p* = 0.001) corroborates biomechanical studies demonstrating that high-momentum antero-posterior vectors simultaneously disrupt the petrous ridge and the pterygomaxillary buttress [17]. Similar associations have been reported in European and North American trauma audits [22], reinforcing the need for integrated craniofacial imaging pathways.

Selection bias: It is important to note that our hospital-based cohort was drawn from two tertiary ENT trauma units. Consequently, milder TBFs managed at peripheral facilities or discharged from emergency departments without imaging are likely under-represented, whereas patients with more severe or complicated injuries are over-represented. This referral pattern helps to explain the relatively high prevalence of facial nerve palsy, otic capsule violation, and associated mid-facial fractures observed in our series.

### 4.2. Clinical Implications

1. Risk stratification: The OCS/OCV schema offers immediate prognostic information; patients with OCV injuries require heightened surveillance for CSF leak, profound hearing loss and delayed facial nerve dysfunction, because these imaging features have been repeatedly associated with—but are not in themselves proof of—higher risks of CSF leak, profound hearing loss, and delayed facial nerve dysfunction [11,21].
2. Early triage of OCV fractures: Because capsule-violating injuries carry substantially higher risks of profound SNHL, acute vestibular dysfunction, and CSF leakage, any HRCT report that identifies OCV geometry should prompt expedited audiologic testing, bedside vestibular assessment, strict head elevation/bed rest, and a low threshold for skull base or neuro-otology consultation [11,13].
3. Imaging algorithms: Given that 27% of our patients had associated mid-facial fractures, a single-session head-and-mid-face HRCT protocol is prudent for moderate-to-severe head trauma, reducing missed diagnoses and additional radiation exposure [13,19].
4. Audiologic management: Early pure-tone audiometry is pivotal for detecting post-traumatic conductive hearing loss—frequently the result of ossicular chain disruption—and for triaging candidates for timely ossicular exploration or reconstruction once patients are stabilized [7,20,23].
5. Facial nerve care: The elevated rate of early palsy mandates systematic electrophysiologic monitoring and, where feasible, prompt decompression for progressive or complete deficits [21].
6. Public health action: Because road traffic accidents account for most injuries in Sudan, targeted road safety legislation and strengthened trauma-care systems are urgently needed to curb RTA-related morbidity [24], including temporal bone complications [25].
7. HRCT red-flag signs: The presence of otic capsule violation, pneumolabyrinth, ossicular chain disruption, or transverse/mixed fracture orientation should prompt early otologic consultation, serial facial nerve assessment, and consideration of multidisciplinary skull base review, even when initial neurologic status is stable [11,13,21].
8. Surgical planning and operative corridors: High-resolution delineation of the otic capsule, promontory, and facial canal course can guide selection of contemporary minimally invasive approaches, such as the transcanal transpromontorial corridor to the internal auditory canal and petrous apex, recently detailed by Molinari et al. [26]. Accurate pre-operative HRCT mapping, therefore, bridges diagnostic imaging with modern skull base surgical strategy.

In daily practice, certain HRCT ‘red-flag’ findings should trigger prompt otologic or skull base review. Table 6 summarizes these imaging signs, the complications they herald, and recommended early management actions.

### 4.3. Study Limitations

Because our statistical analyses were exploratory and limited to univariate tests, the observed associations cannot be considered as independent predictors of outcome. We acknowledge that β_2_-transferrin testing was unavailable for most participants; therefore, cerebrospinal fluid (CSF)–leak status relied primarily on clinical observation and HRCT surrogates, which may have led to under- or overestimation. Likewise, three patients could not undergo formal pure-tone audiometry because of young age (n = 2) or clinical instability (n = 1); their exclusion from quantitative audiologic analyses introduces a modest risk of selection bias. Several limitations temper interpretation. Hospital-based recruitment at two tertiary ENT trauma centers may have preferentially captured patients with more complex or severe injuries, thereby inflating the apparent prevalence of facial nerve palsy and other complications and limiting the generalizability of our findings to community or primary-care settings. The cross-sectional design prevents assessment of long-term audiologic or neurologic recovery. Advanced MRI, which could refine the detection of labyrinthine or neural injury, was unavailable. Inter-observer agreement for key imaging variables was assessed and found to be substantial to almost perfect (κ = 0.77–0.92), as reported in Section 3.5; however, our study did not evaluate intra-observer repeatability, which remains a topic for future work. The high facial palsy prevalence should therefore be interpreted in light of (i) our inclusive HB ≥ II case definition, (ii) the early timing of assessment when transient neuropraxia cannot be excluded, and (iii) tertiary referral selection bias.

In addition, the relatively small cohort size inevitably led to low cell counts in several 2 × 2 comparisons. As a consequence, a number of the crude odds ratios we reported are accompanied by wide 95% confidence intervals, indicating limited statistical precision. These estimates should therefore be interpreted with caution and viewed as hypothesis-generating rather than definitive measures of effect size. Larger, multicenter datasets will be required to obtain narrower confidence bounds and permit multivariable adjustment.

### 4.4. Conclusions

HRCT offered a reliable descriptive characterization of temporal bone fracture orientation and otic capsule integrity in this cross-sectional cohort. Secondary analyses indicated that, within our sample, transverse/mixed and capsule-violating fractures were more frequently accompanied by complex mid-facial fractures and showed higher rates of early conductive hearing loss and facial nerve palsy.

### 4.5. Recommendations

1. Implement standardized thin-slice HRCT protocols that include temporal bone reconstructions for all patients with moderate-to-severe head or mid-facial trauma.
2. Develop multidisciplinary trauma pathways involving radiology, otology, maxillofacial surgery, and neurosurgery to expedite management of OCV and complex craniofacial fractures.
3. Enhance public-health measures—particularly road safety legislation and enforcement—to curb high-energy RTAs.
4. Undertake prospective, multicenter studies with larger cohorts and longitudinal follow-up to refine prognostic models and evaluate functional outcomes in sub-Saharan Africa.

## Figures and Tables

**Table 1:** Demographic characteristics and mechanism of injury (n = 45).

Variable	Category	n	%
Age, years (mean ± SD)	35.9 ± 17.4	–	–
Age group	<18	4	8.9
	18–35	21	46.7
	36–50	13	28.9
	51–65	4	8.9
	>65	3	6.6
Sex	Male	35	77.8
	Female	10	22.2
Laterality	Left	26	57.8
	Right	17	37.8
	Bilateral	2	4.4
Mechanism	Road traffic accident	26	57.8
	Interpersonal blow	8	17.8
	Fall	5	11.1
	Gunshot wound	4	8.9
	Work-related	2	4.4

**Table 2:** Presenting symptoms, otoscopic/neurologic signs, and audiometric outcomes (n = 45).

Symptom/Sign	n	%
Decreased hearing	36	80.0
Otorrhoea (any)	20	44.4
-- Bloody	9	20.0
-- CSF	4	8.9
Tinnitus	19	42.2
Otalgia	15	33.3
Vertigo	15	33.3
Aural fullness	4	8.9
Facial nerve palsy (HB ≥ II)	26	57.8
External auditory canal laceration	16	35.6
Tympanic membrane perforation	22	48.9
Hemotympanum	16	35.6

Other auricle injuries include simple laceration, mastoid bruising, hematoma, and partial amputation. HB = House–Brackmann; PTA = pure-tone audiometry.

**Table 3:** HRCT findings of temporal bone fractures (n = 45).

HRCT Variable	n	%
Fracture orientation		
-- Longitudinal	27	60.0
-- Transverse	9	20.0
-- Mixed/oblique	9	20.0
Otic capsule status		
-- Sparing (OCS)	33	73.3
-- Violating (OCV)	12	26.7
Ossicular chain disruption	8	17.8
Tympanic plate fracture	6	13.3
Pneumolabyrinth/CSF leak	4	8.9

“Longitudinal,” “transverse,” and “mixed/oblique” are mutually exclusive fracture planes whose percentages sum to 100% of the cohort (n = 45). Otic do not traverse the bony labyrinth, whereas otic capsule-violating (OCV) indicates cochleo-vestibular involvement; these categories are likewise mutually exclusive. Ossicular chain disruption, tympanic plate fracture, and pneumolabyrinth/CSF leak represent additional, non-mutually exclusive HRCT findings, so their percentages can exceed 100%. All percentages are calculated from the total sample (n = 45) and rounded to one decimal place.

**Table 4:** Associated craniofacial fractures detected on HRCT (n = 45).

Fracture Type	n	%
None	33	73.3
Lefort I	1	2.2
Lefort II	4	8.9
Lefort III	7	15.6

No isolated zygomatic, orbital floor, or mandibular fractures were observed.

**Table 5:** Association between fracture orientation (longitudinal vs. transverse/mixed) and selected predictors (n = 45).

Predictor	Test Statistic	df	*p*-Value ^1^
Age group (<18, 18–35, 36–50, 51–65, >65 yr)	χ^2^ = 6.1	4	0.21
Sex (male/female)	χ^2^ = 5.1	2	0.08
Concomitant craniofacial fracture (present/absent)	χ^2^ = 16.2	2	**0.001**

^1^ Pearson’s chi-square test; *p* < 0.05 is considered statistically significant (bold).

**Table 6:** Key HRCT findings and their immediate clinical implications.

HRCT Red Flag Finding	Typical Imaging Appearance	Principal Clinical Concern(s)	Recommended Early Action
Otic capsule violation	Fracture line traversing cochlea/vestibule; lucency or sclerosis; ± pneumolabyrinth	Profound SNHL; perilymph fistula or CSF leak; delayed FN palsy	Admit with head elevation and bed rest; serial audiometry and FN checks; early skull base or neuro-otology consultation
Ossicular chain disruption	Widened or displaced ossicular joints; absent or mal-aligned ossicles	Conductive hearing loss; risk of chronic otitis media or ossicular erosion	Formal PTA; arrange otologic follow-up; consider exploratory tympanotomy/ossiculoplasty
Tympanic plate fracture	Intracochlear/intravestibular air; air–fluid level in the middle ear/mastoid	Active perilymph or CSF leak; meningitis risk	Bed rest; prophylactic antibiotics per protocol; neurosurgical input if leak persists
Pneumolabyrinth or frank CSF in the middle ear	Air within vestibule/cochlea; air–fluid level in tympanum	Active perilymphatic or CSF leak → meningitis risk	Bed rest, prophylactic antibiotics per protocol, neurosurgical input if persistent
Facial canal fracture/dehiscence	Cortical break along the labyrinthine or Cortical break along the labyrinthine, tympanic, or mastoid segment of the FN canal	Immediate or delayed facial-Immediate or delayed FN palsy	Serial HB grading; EMG if HB ≥ III > 72 Serial HB grading; facial nerve EMG ≥ 72 h if HB ≥ III; consider surgical decompression

Abbreviations: SNHL—sensorineural hearing loss; FN—facial nerve; PTA—pure-tone audiometry; EAC—external auditory canal; HB—House–Brackmann grade; CSF—cerebrospinal fluid.

## Data Availability

The data presented in this study are available from the corresponding author upon reasonable request. The dataset contains individual patient-level clinical and imaging information; open release is therefore precluded by institutional ethics-board policy and national privacy regulations.

## Supplementary Materials

The following supporting information can be downloaded at: https://www.mdpi.com/article/10.3390/diagnostics16050718/s1, Table S1: Crude odds ratios (ORs) with 95% confidence intervals (CIs) for selected 2 × 2 comparisons (exploratory analysis); Table S2: Otic-capsule status versus early functional outcomes (n = 45).

## References

[1] Henry M., Hern H.G. (2019). Traumatic injuries of the ear, nose, and throat. Emerg. Med. Clin. N. Am..

[2] Parsons M., Policeni B., Juliano A., Agarwal M., Benjamin E.E., Burns J., Doerr T., Dubey P., Friedman E.R., Gule-Monroe M.K. (2022). ACR Appropriateness Criteria^®^ Imaging of Facial Trauma Following Primary Survey. J. Am. Coll. Radiol..

[3] Ravindran D., Dev S.S., Sindhu B.S. (2024). Otological manifestations of temporal bone fractures. J. Med. Sci. Res..

[4] Shih R., Burns J., Ajam A., Broder J., Chakraborty S.S., Kendi A., Lacy M.E., Ledbetter L.N., Lee R.K., Liebeskind D.S. (2021). ACR Appropriateness Criteria^®^ Head Trauma: 2021 Update. J. Am. Coll. Radiol..

[5] Keshavamurthy V.B., Ajith K.M., Maradi N., Gupta R., Jain S. (2022). Correlation of hearing outcome in otic capsule sparing temporal bone fractures using temporal bone sub-site classification: A cross-sectional descriptive study. Egypt. J. Otolaryngol..

[6] Yang N. (2023). Fracture of the Petrous Carotid Canal. Philipp. J. Otolaryngol. Head Neck Surg..

[7] Juliano A.F. (2018). Cross-sectional imaging of the ear and temporal bone. Head Neck Pathol..

[8] Tuan N., Khoa L., Bao N., Tu P., Phuoc L. (2023). Endovascular management of giant post-traumatic pseudoaneurysm in cavernous sinus: A case report. Radiol. Case Rep..

[9] Vahidi N., Wang W., Lee T., Inman J., Ducic Y. (2019). Medicolegal Aspects of Craniofacial Trauma. Facial Plast. Surg..

[10] Molnár D., Mező M., Vaska Z., Sevecsek Z., Helfferich F. (2021). Report of an Otic Capsule Disrupting Fracture of the Temporal Bone: Visualization of Pneumolabyrinth and Functional Assessment. Cureus.

[11] Singh R., Mishra G., Patwa P., Dhande R., Gowda H., Singh S., Goyal A.V., Bele A. (2022). Correlation of Temporal Bone Fracture on Computed Tomography Scan with Hearing Loss in Posttraumatic Patients. J. Datta Meghe Inst. Med. Sci. Univ..

[12] Jongbloed W., Campbell D., Kuo C., Zhong K., Cavanagh N. (2025). A Five-Year Review of Temporal Bone Fractures at a Level One Trauma Center and Examination of the Impact of the COVID-19 Pandemic. Surgeries.

[13] Wu C., He Q. (2025). Audiometric and vestibular outcomes following temporal bone fractures: A retrospective analysis of a major trauma center cohort in China. Front. Med..

[14] Shapira U., Klein L., Oron Y., Handzel O., Abu-Eta R., Muhanna N., Shilo S., Brenner A., Ungar O.J. (2024). The Role of Temporal Bone Pneumatization on Fracture Line and Involved Cranial Structures. Otolaryngology.

[15] Wood C.P., Hunt C.H., Bergen D.C., Carlson M.L., Diehn F.E., Schwartz K.M., McKenzie G.A., Morreale R.F., Lane J.I. (2014). Tympanic plate fractures in temporal bone trauma: Prevalence and associated injuries. Am. J. Neuroradiol..

[16] Ghiasi S., Banaei M. (2016). Bilateral facial paralysis caused by temporal bone fracture: A case report. Arch. Trauma Res..

[17] Huang L.K., Tu H.F., Jiang L.D., Chen Y.Y., Fu C.Y. (2019). Evaluation of concomitant orbital-floor fractures in patients with head trauma using conventional head CT: A retrospective study at a level II trauma centre. J. Clin. Med..

[18] Koo T.K., Li M.Y. (2016). A guideline of selecting and reporting intraclass correlation coefficients for reliability research. J. Chiropr. Med..

[19] Shokry M., Sameh S., Raghib M., Sayed S., Mohamed A. (2022). Accuracy of multislice computed tomography scan in cases with facial nerve paralysis due to temporal bone trauma. Egypt. J. Neck Surg. Otorhinolaryngol..

[20] Deshmukh K.A., Fatima U., Siddiqui A., Tegnoor M.S. (2023). Evaluation and outcomes of hearing loss in temporal bone fractures: A prospective study. Cureus.

[21] Gordin E., Lee T.S., Arnaoutakis D. (2015). Facial-nerve trauma: Evaluation and considerations in management. Craniomaxillofacial Trauma Reconstr..

[22] Kang T., Ha R., Oh J., Sunwoo W. (2019). The potential protective effects of temporal bone pneumatization: A shock absorber in temporal bone fracture. PLoS ONE.

[23] Aljehani R., Al-Shamani A.N. (2019). A tale of 1 year: A case of bilateral conductive hearing loss due to bilateral ossicular chain disruption post head trauma. J. Surg. Case Rep..

[24] Bali R.K., Sharma P., Garg A., Dhillon G. (2013). A comprehensive study on maxillofacial trauma conducted in Yamunanagar, India. J. Inj. Violence Res..

[25] Sasindran V., Joseph A., Babu B., George P. (2014). A case of posttraumatic incudomalleolar disruption. Indian J. Otol..

[26] Molinari G., Chiari F., Presutti L., Fermi M., Fernandez I.J., Alicandri-Ciufelli M. (2022). Expanded transcanal transpromontorial approach for acoustic neuroma removal. Laryngoscope.

