# Correction: Edris et al. Temporal Bone Fractures on High-Resolution CT: Bridging Radiologic Detail with Otologic Anatomy and Surgical Implications. *Diagnostics* 2026, *16*, 718

**DOI:** 10.3390/diagnostics16091399

**Published:** 2026-05-06

**Authors:** Osama M. K. Edris, Abdulgaffar Bashir Adam, Emad Ali Albadawi, Ahmad Mahroos ALGhabban, Razan Saad M. Alqarni, Wejdan Hussain Owaydhah, Omar A. Alharthi, Eyad Khattab, Fahd Alharbi, Yasir Hassan Elhassan

**Affiliations:** 1 Department of Otolaryngology, Faculty of Medicine, University of Khartoum, Khartoum 11115, Sudan; osamamk@yahoo.com; 2 Khartoum ENT Teaching Hospital, Khartoum 11112, Sudan; 3 Department of Basic Medical Sciences, College of Medicine, Taibah University, Madinah 42353, Saudi Arabia; 4 Department of Internal Medicine, College of Medicine, Taibah University, Madinah 42353, Saudi Arabia; 5 Department of Emergency Medicine, King Saud University Medical City, Riyadh 42353, Saudi Arabia; 6 Department of General and Specialized Surgery, College of Medicine, Taibah University, Madinah 42353, Saudi Arabia

In the original publication [1], there is an error in Figure 1. Figure 1 had an incorrect temporal bone with a mislabeled “Tympanic part” and wrong coloring. The corrected Figure 1 appears below. The authors state that the scientific conclusions are unaffected. This correction was approved by the Academic Editor. The original publication has also been updated.

## Figures and Tables

**Figure 1:**
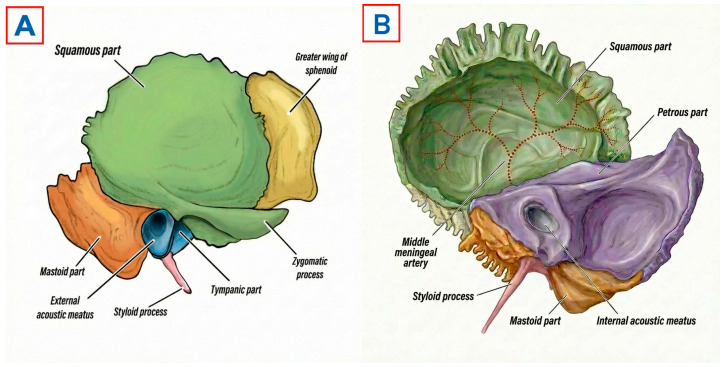
Anatomy of the right temporal bone. (**A**) Lateral external (otologic) surface. (**B**) Medial intracranial surface.
