# Supplementary material for: Temporal Bone Fractures on High-Resolution CT: Bridging Radiologic Detail with Otologic Anatomy and Surgical Implications
Source: Diagnostics (Basel). 2026 Feb 28;16(5):718. doi: 10.3390/diagnostics16050718 (PMC12984264; doi:10.3390/diagnostics16050718)
Supplement: Supplementary file 1 [file diagnostics-16-00718-s001.zip › diagnostics-4124799-supplementary.pdf]

Supplementary Table S1. Crude odds ratios (ORs) with 95 % confidence intervals (CIs) for selected 2 × 2 comparisons (exploratory analysis).

**Exposure / Outcome Comparison***	**OR (95 % CI)**
Non-longitudinal (transverse + mixed) vs. longitudinal fracture → Le Fort II–III present	8.4 (2.0–34.6)
Otic-capsule-violating vs. sparing → CSF leak present	5.1 (0.8–33.6)
Otic-capsule-violating vs. sparing → Facial-nerve palsy (HB ≥ II)	3.6 (1.0–13.0)
Non-longitudinal vs. longitudinal → Conductive hearing loss present	1.2 (0.3–5.5)

*All odds ratios are unadjusted and derived from 2 × 2 contingency tables; HB = House–Brackmann grade.

Supplementary Table S2. Otic-capsule status versus early functional outcomes (n = 45)

**Otic-capsule status**	**n**	**Conductive HL (CHL) n (%)**	**Sensorineural HL (SNHL) n (%)**	**Mixed HL n (%)**	**Facial-nerve palsy (HB ≥ II) n (%)**
OCS (sparing)	33	26 (78.8)	1 (3.0)	6 (18.2)	16 (48.5)
OCV (violating)	12	5 (41.7)	5 (41.7)	2 (16.6)	10 (83.3)
**p-value***	–	**0.004**			**0.028**

*Fisher's exact test for the distribution of (i) hearing-loss type (CHL / SNHL / mixed) and (ii) facial-nerve palsy across otic-capsule-sparing (OCS) versus otic-capsule-violating (OCV) fractures.

Note: All hearing-loss percentages are calculated within each otic-capsule stratum. Facial-nerve-palsy figures combine immediate- and delayed-onset cases, as defined in Section 2.4 of the main text.
